# Mechanical Properties of a Bone-like Bioceramic–Epoxy-Based Composite Material with Nanocellulose Fibers

**DOI:** 10.3390/ma16020739

**Published:** 2023-01-12

**Authors:** Young-Seong Kim, Jin Woo Baek, Zhengyun Jin, Hee Chang Jeon, Min-Woo Han, Joong Yeon Lim

**Affiliations:** 1 Department of Mechanical, Robotics and Energy Engineering, Dongguk University, Seoul 04620, Republic of Korea; 2 Quantum Functional Semiconductor Research Center, Dongguk University, Seoul 04620, Republic of Korea

**Keywords:** bone-like composite materials, hydroxyapatite, zirconia oxide, cellulose nanocrystals, nano network, linear elastic modulus, stiffness

## Abstract

Several composite materials are being investigated as reinforcement fillers for surgery simulations. This study presents an artificial composite material with properties similar to those of the human bone, which may be used in surgery simulations. Moreover, considering the potential toxicity of debris generated during sawing, a safe epoxy-based composite material was synthesized using cellulose nanocrystals (CNCs) and bioceramics (i.e., hydroxyapatite, Yttria stabilized zirconia oxide, Zirconia oxide), which were used to mimic the stiffness of human bone. To examine the change in mechanical properties according to the composition, 1, 3, and 5 wt% of CNCs were mixed with 5 wt% of the bioceramics. When CNCs were added at 1 wt%, there was a confirmed change in the non-linear stiffness and ductility. The CNC-added specimen fractured when forming a nano-network around the local CNCs during curing. In contrast, the specimen without CNCs was more densely structured, and combined to form a network of all specimens such that a plastic region could exist. Thus, this study successfully manufactured a material that could mimic longitudinal and transverse characteristics similar to those of real human bone, as well as exhibit mechanical properties such as strength and stiffness. Bioceramics are harmless to the human body, and can be used by controlling the added quantity of CNCs. We expect that this material will be suitable for use in surgery simulations.

## 1. Introduction

Surgeons perform simulations for bone cutting or drilling, which has been the focus of many studies. In addition to mechanical properties, studies on the heat generated during bone cutting and drilling have also been performed [1,2,3].

Typically, elderly patients with bone disease undergo surgery to cut out the diseased bones and install implants; such surgeries require extensive training and practice by orthopedic surgeons. In fact, it has been demonstrated that surgical residents can improve their memory and technical skills required for surgical procedures through training simulations [4].

Although cadaver bones can be used in surgical education and training, they are a suboptimal solution because of the associated costs, restrictions on usable fields, and challenges with preservation. Therefore, epoxy-based composites are being used as alternatives. Several composite materials are being employed as reinforcement fillers, and extensive research is being conducted on composite materials with properties similar to those of the human bone. Additionally, studies are being conducted to develop composite materials with the same mechanical properties as bone and to use them for real bone repair [5,6]. Epoxy resins can facilitate the implementation of anatomically correct and detailed shapes, including the surface of cortical bone, using a mold of the desired bone shape [7]. Furthermore, epoxy-based composites can be used to develop consistent models because there is a negligible change in shape after the completion of curing. Thus, as traditional surgical education has many limitations [8], there is wide interest in safe, cost-effective, and anatomically and mechanically similar models as the human bone [7,9].

Some researchers have attempted to mimic actual surgeries using virtual-reality- and augmented-reality-based simulation technologies [10]. Furthermore, there are several bone-machining studies on the generation of debris or cutting forces [11,12,13,14,15]. Debris is inevitably generated when composite materials with properties similar to those of the human bone are cut or drilled during a simulation [11]. Hence, the potential hazard posed by the possible penetration of these pieces into the skin should be considered when designing composites using synthetic fibers [16,17]. A suitable ventilation system and other safety tools are required to work with these materials. Glass fibers and carbon fibers have also been used to provide properties similar to those of the human bone [18]; however, these materials have their own disadvantages and have posed safety risks in the surgical work environment of surgeons owing to their debris. Similarly, synthetic-fiber-based composite materials have been tested and found to pose similar hazards [16,17]. For example, synthetic fibers can cause dermatitis by penetrating the skin during machining. In particular, aerosols generated during bone cutting are highly hazardous to health [11]. In studies conducted on actual industrial workers, it was reported that dermatitis occurred primarily on the hands and arms [19,20].

The bone model must also reproduce the strength and stress–strain characteristics of human bone. According to experimental studies, the human femur bone in men and women has average tensile strengths of 39.74 and 30.08 MPa, respectively; average compressive strengths of 141.6 and 118.91 MPa, respectively; and Young’s moduli of 338.3 and 404.7 MPa, respectively [21]. However, the bone strength and modulus change with age, while the mechanical properties also change with disease. For example, in osteoarthritis, the yield strength increases and toughness decreases, and in osteoporosis, both the yield strength and toughness decrease [22]. In addition, because of the anisotropic properties of bone, i.e., as the bone strength depends on the direction [23], a composite material that can express various mechanical properties depending on the angle requirement in the surgical training, which can vary from longitudinal to transverse, is needed. In other words, artificial bone materials with adjustable bone strengths and physical characteristics that can be tuned according to the disease, age, sex, and direction of the cutting plane are needed as a surgical educational tool. The materials should also incorporate bioceramics to be safe and cost-effective for surgical trainees. In essence, such a bone would be a suitable substitute for a cadaver or other bone models.

However, since epoxy exhibits a non-linear stress–strain curve for all load regions [24], it is difficult to express the bilinear characteristics of the typical stress–strain curve as well as the monotonically increasing nature of compact bone [25]. Therefore, it is necessary to develop a material that: (i) meets the strength requirements of the femur bone [21], (ii) reproduces the typical stress–strain curve of compact bone, and (iii) does not release harmful debris during machining.

This study aims to present the orthopedic surgeons with an artificial-bone material that does not pose a health and safety hazards in simulation-operations, such as the penetration of sawing debris into the skin. In other words, the currently used composite materials are made of synthetic fibers have a disadvantage that the fibers penetrate the skin during machining [16,17]. We eliminate this problem by developing a novel material that would have stress–strain characteristics similar to those of the real human bones [25]. Therefore, we tried to match the linear stress–strain curve characteristics, which is an important feature, with the same strength as the real bone through the use of an epoxy-based composite material. To match the linear stiffness and brittleness through linear stress–strain characteristics, we reproduced the mechanical and safety properties by creating a network-like structure in a polymer with an addition of CNC.

In this study, we used composite materials based on hydroxyapatite (HAP), zirconia oxide (ZO), and yttria-stabilized zirconia oxide (YZO), which are currently used in the biomedical field. These materials can increase the strength and elastic modulus of epoxy, as well as control various mechanical properties. These materials also provide a linear stress–strain curve, have a harmless and non-toxic composition, and can be used to produce surgical training bones of various strengths, similar to real bone. Furthermore, these materials do not pose issues such as the likelihood of the sawing debris penetrating the skin when trainees cut or drill through the artificial bones during orthopedic surgery training. Of these materials, HAP has the most similar chemical composition to bone and teeth [26,27,28,29], ZO shows improved strength and stiffness [30], and YZO shows excellent fracture strength [31]. To match the stress–strain curve of the synthesized material to that of real bone (i.e., in terms of the linear stiffness and brittleness), cellulose nanocrystals (CNCs) were used as an additive to maintain the mechanical properties and safety characteristics. In previous studies, it was shown that the strength decreased when CNCs were added at more than 1 wt%, which also made the composite brittle [32]. Therefore, we focused on how excess CNC addition to epoxy can affect the elastic modulus of epoxy through the generation of a network system [33] and how nanoparticles embrittle epoxy-based composite materials [34,35]. In order to analyze the mechanical properties of the composite materials, a tensile test and a compression test were conducted according to ASTM standards, and the Scanning electron microscopy (SEM) images of the particles generated by cutting were analyzed by Image J software(version 1.53t). To verify the differences in mechanical properties, we conducted an analysis of variance (ANOVA) for a total of six groups: HAP/YZO/ZO/HAP+CNC/YZO+CNC/ZO+CNC.

## 2. Materials and Methods

### 2.1. Materials

The epoxy resin (product number: 105) and hardener (product number: 207) were obtained from West System Co. (Bay City, MI, USA). Hydroxyapatite (Cas 1306-06-5, purity ≥ 90%, product number:21223), zirconium oxide (Cas 1314-23-4, purity ≥ 99%, diameter 5 um, product number:230693), and yttria-stabilized zirconium oxide (Cas 114168-16-0, purity ≥ 99.9%, submicron, ~8 wt.% yttria as stabilizer, product number: 464228) in the powder form were purchased from Sigma Aldrich (St. Louis, MO, USA). CNCs (aqueous gel form (10.4 wt%)) were obtained from the Process Development Center of the University of Maine. The specimens were fabricated using a silicon mold (Mold Max 30, Smooth-on, Inc., Macungie, PA, USA).

### 2.2. Preparation of Nanocellulose

The CNCs (aqueous gel form, 10.4 wt%) was purchased from the University of Maine (the university sells this product). Thus, the CNC powders were fabricated by freeze-drying the aqueous gel for three days using a freeze dryer (ilShinBioBase Co., Ltd., Dongducheon, Republic of Korea, FD8508) in a vacuum of 5 mTorr at −80 °C.

### 2.3. Preparation of Composite Materials

**Figure 1:**
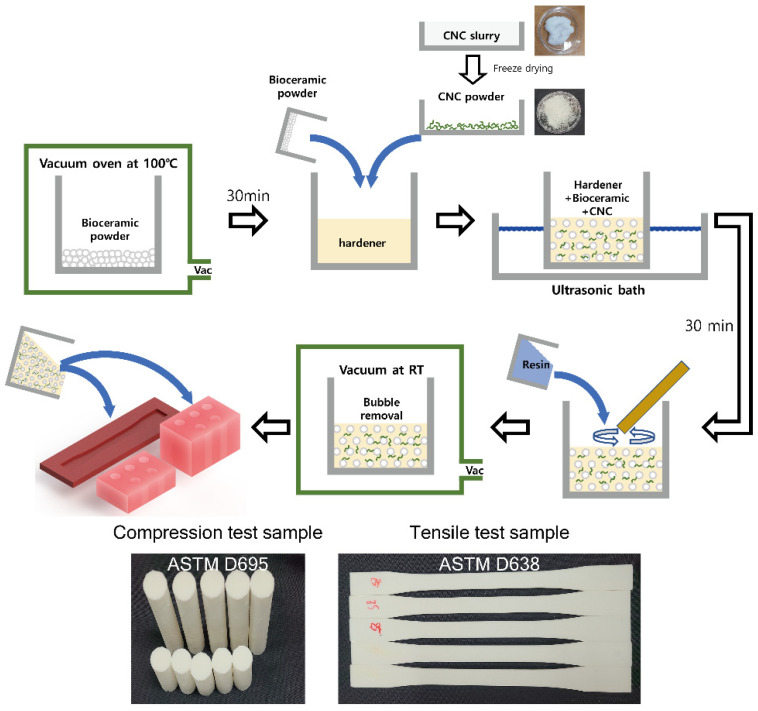
Synthesis of composite material.

First, to remove moisture from the purchased bioceramic, it was vacuum-dried at 100 °C for 30 min and then used for the material synthesis. Subsequently, the required amount of bioceramic was measured and dried according to the wt% of each specimen. Next, the hardener was added, and the specimen was sonicated for 30 min to ensure that the hardener and bioceramic powder were sufficiently mixed. After ultrasonic treatment (Ultrasonication: 42 kHz, 135 W at room temperature), the resin was poured and mixed. To remove air bubbles, the mixture was vacuum-dried at room temperature and poured into a silicone mold (Figure 1). After curing for one day, the specimens were demolded and cured at room temperature for two weeks. The hardener and resin were mixed at a weight ratio of 1:3, and the composite materials’ weight ratio is in Table 1. Subsequently, the CNCs were added into the mixture, which was then sonicated. CNCs were added along with the bioceramic to the hardener for ultrasonic treatment, and finally, the resin was poured into the hardener.

### 2.4. Material Characterization

A tensile test was conducted on the fabricated type-I specimens in accordance with ASTM D638. The specimen for the tensile test was dog-bone-shaped. As shown in Figure 1, the type 1 specimen has a width of 13 ± 0.5 mm and thickness of 3.2 ± 0.4 mm. The tensile test was conducted at a speed of 5 mm/min. A compression test was conducted in accordance with ASTM D695. The strength was measured using a cylindrical specimen with a diameter and height of 12.7 and 25.4 mm, respectively. To measure the elastic modules, a specimen was fabricated separately with a diameter and height of 12.7 and 50.8 mm, respectively. The test was conducted at a speed of 1.3 mm/min. Analysis was performed using a universal testing machine (Oriental TM Co., Ltd., Siheung, Republic of Korea, OTU-2) and the load cell for measuring the tension and compression employed in UTMs was used. The load cell had a capacity of 2000 kgf (Bongshin Load Cell Co., Osan, Republic of Korea). SEM was performed after cutting the compression-tested specimen using a precision band saw machine (Woosung E&I Co., Ltd., Seongnam, Republic of Korea, MBS500) and carbon-coating (sputtering for 60 s) the corresponding plane. SEM images of the particles were analyzed with Image J software to measure each particle size using the scale bar; in all, 73 particles were measured for HAP–epoxy and 52 particles for HAP–epoxy with CNCs.

## 3. Results and Discussion

**Figure 2:**
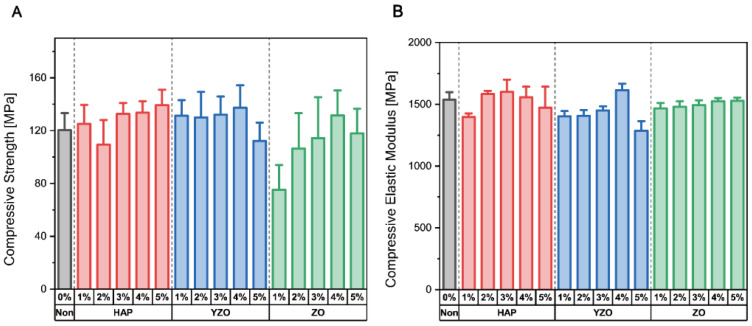
Results of the compression test on different types of bioceramic–epoxy composite materials. (**A**) Compressive strength. (**B**) Compressive elastic modulus.

In this study, an epoxy-based composite was fabricated using CNCs and three bioceramics, namely HAP, YZO, and ZO, as reinforcements. The composite showed different trends of the compressive strength change according to the wt% of the bioceramics. HAP provided the greatest strength increase at 5 wt%, and its average strength was 139.23 MPa, higher than that of ordinary epoxy by 15.62%. YZO provided a stable strength without significant changes by wt%. ZO had the highest strength of 131.5 MPa at 4 wt%, which increased by 9.2% (Table 2 and Figure 2A). The measured compressive elastic modulus did not significant differ from that of ordinary epoxy (Table 2 and Figure 2B). In the case of the compressive elastic modulus in Table 2, the bioceramic HAP, YZO, and ZO showed a slight increase in strength with wt%. The measured compressive elastic modulus did not significantly differ from that of ordinary epoxy (Table 2 and Figure 2B). The compressive strength and compressive elastic modulus were calculated using a universal testing machine (UTM). The compressive strength value was calculated as follows: compression force divided by the area at maximum load. The modulus was calculated using the slope at the start of the measurement.

**Figure 3:**
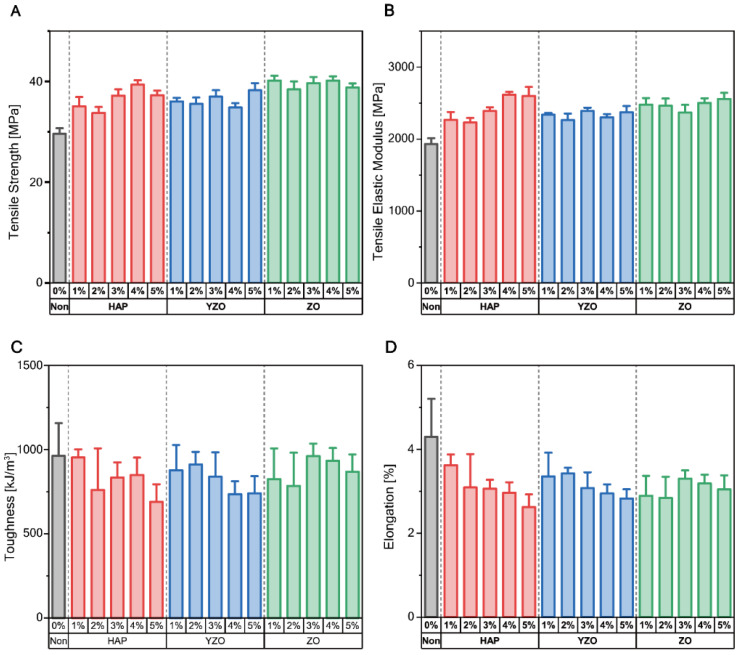
Results of the tensile test of different types of bioceramic–epoxy composite materials. (**A**) Tensile strength. (**B**) Elastic modulus. (**C**) Toughness. (**D**) Elongation.

When only bioceramic was added (i.e., without the CNC), the largest increases in tensile strength with HAP, ZO, and YZO were 32.93%, 35.74%, and 29.05%, respectively, which correspond to 39.39 MPa at 4 wt%, 40.22 MPa at 4 wt%, and 29.05% at 5 wt%, respectively. Thus, it can be observed that changes according to the wt% of bioceramics are not large (Figure 3A). Elastic modulus (stiffness) showed the largest change with HAP, where the stiffness was directly proportional to the weight percentage. Specifically, when HAP was used in the synthesis at 4 wt%, the elastic modulus increased by 35.48% to 2617.47 MPa (Figure 3B). YZO provided a greater elastic modulus than ordinary epoxy, but no significant change could be observed as the weight percentage increased. ZO provided a higher elastic modulus than other specimens at low wt%, but the modulus did not change significantly despite the addition of 5 wt% (Table 3). In HAP, as the wt% increased, the compressive strength was the highest (Table 2), and the tensile elastic modulus also showed the highest value. In addition, the toughness also continuously decreased, exhibiting a brittle change at 5 wt% (Table 3). The bone should be able to exhibit various mechanical properties according to age and gender, but in the case of HAP, various mechanical properties of bones can be expressed by such changes in compression and tensile material properties.

Evidently, the elastic moduli of HAP–epoxy composites increase with a similar tendency as the yield strength.

The toughness of the epoxy composite decreased with the addition of HAP. At the highest concentration of HAP (5 wt%), toughness decreased to 689.28 kJ/mm^3^ (Figure 3C). Moreover, elongation also decreased (Figure 3D), and a reduction in the plastic region can be observed in the graph.

To examine the effect of CNCs and bioceramics in the epoxy-based composite, 1, 3, and 5 wt% of CNCs were mixed with 5 wt% of bioceramic, and the changes in their properties were analyzed.

**Figure 4:**
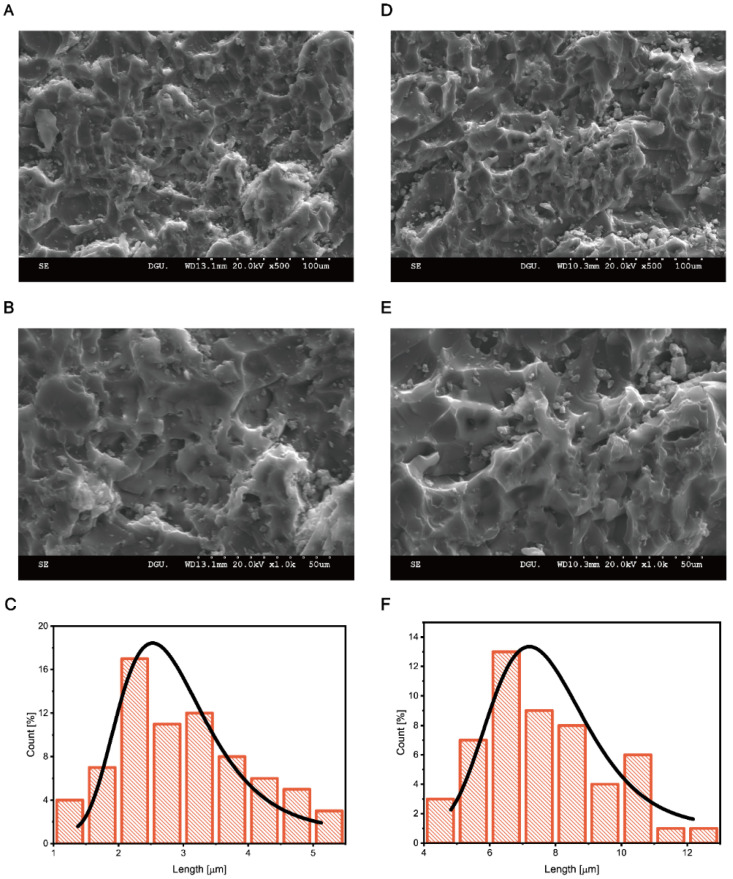
Scanning electron microscopy (SEM) images. (**A**) HAP–epoxy composite materials. (**B**) An enlarged image of (**A**). (**C**) The particle size of HAP–epoxy composite materials after sawing. (**D**) HAP–epoxy composite with CNC. (**E**) An enlarged image of (**D**). (**F**) The particle size of HAP–epoxy composite with CNC after sawing. (**C**,**F**) are statistics of particle size from SEM images with a total area of 0.0439 mm^2^.

CNC formed a network-like chain and hardened the resultant mixture. It was nano-sized, and as the concentration of CNC increased, it became denser and more concentrated; consequently, it easily crumbled. With increasing concentration, only the strength decreased, while the characteristics of the elastic region were maintained. These characteristics can be observed more clearly in the SEM image of a cross-sectional cut (performed with a saw) (Figure 4).

**Figure 5:**
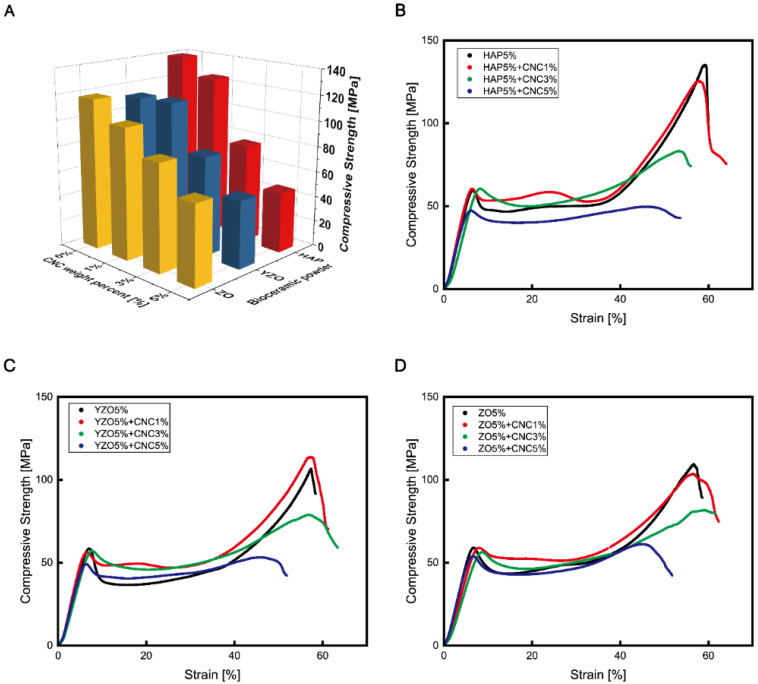
(**A**) Comparison of compressive strength vs. strain changes according to CNC weight percentage in each bioceramic–epoxy composite material. (**B**) Compressive strength vs. strain in HAP–epoxy composite material as function of CNC content. (**C**) Compressive strength vs. strain in YZO–epoxy composite material as a function of CNC content. (**D**) Compressive strength vs. strain in ZO–epoxy composite material as a function of CNC content.

When the specimen was cut using an electric saw, the HAP-only specimen featured small particles of ~5 µm or less, and the cut surface was smooth (Figure 4A,B). However, the specimen with CNC exhibited a rough cutting surface and the formation of numerous particles with larger particle sizes (~12 µm) (Figure 4D,E). This characteristic can be observed for ZO and YZO composites as well, which agrees well with the trend observed in Figure 5 below. It is expected that a network-like structure in a CNC-added polymer would be able to match the mechanical properties of the stiffness and toughness of real bones, which would be adjustable according to the concentration of CNCs in the composite. In other words, a network-like structure can be formed by the particles around CNC. Because the strength required to achieve cracking can be controlled by adjusting the size of internal particles, an epoxy-based composite of this type is expected to have a linear elastic modulus and brittleness that are similar to those of real bone.

When the sizes of particles in the SEM images were measured, apparent differences could be observed. A comparison of the lengths of particles shows that the particle size was in the range of 1–5 µm for the composite with HAP (Figure 4C) and 4–12 µm in the composites with HAP–CNC (Figure 4F). This confirms that the particle size increased owing to the nano-networks around CNC.

Evidently, the change in compressive strength was insignificant at 1 wt%. In the case of YZO, the compressive strength was higher with CNC. The strength decreased at 3 and 5 wt%, and when fracture occurred, the specimen was slowly fractured in a ductile-like manner instead of brittle fragmentation (Figure 5). When the tested specimens were compared, ordinary epoxy specimens and specimens containing bioceramics exhibited different compression fracture patterns. However, the specimens with CNC fractured by generating large particles that were similar to bone chips, which occurred when the bone was cut and as the compressed areas spread widely [36].

**Figure 6:**
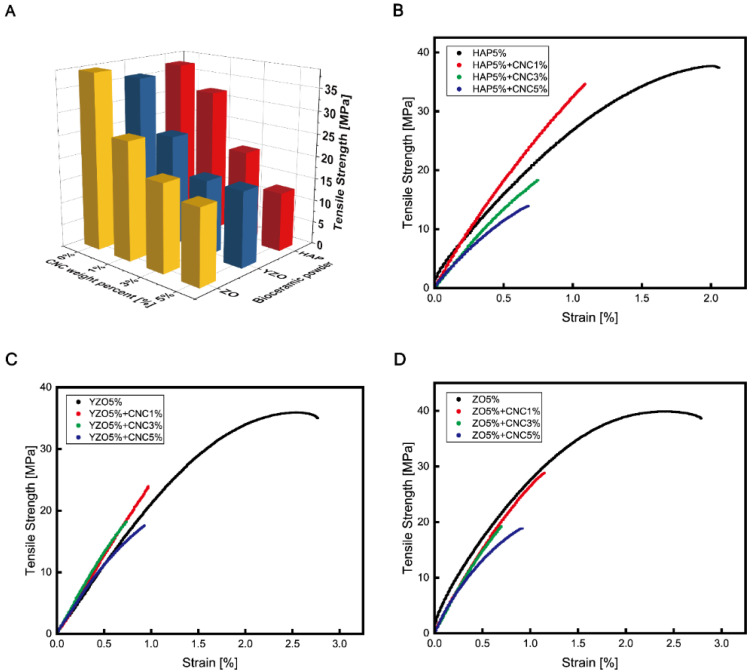
(**A**) Comparison of tensile strength change according to CNC weight percentage in each bioceramic–epoxy composite material. (**B**) Tensile strength vs. strain in HAP–epoxy composite material as a function of CNC content. (**C**) Tensile strength vs. strain in YZO–epoxy composite material as a function of CNC content. (**D**) Tensile strength vs. strain in ZO–epoxy composite material as a function of CNC content.

In the tensile test, the strengths and elongations of all three bioceramics decreased as the number of CNCs increased. Moreover, only the elastic region remained, whereas the plastic region disappeared (Figure 6). YZO and ZO provided no significant change in strength at 3 wt% and 5 wt%, but the strength of HAP-CNC continued to decrease as the weight percentage of CNC increased (Figure 6A).

For the HAP–CNC composite, strength and elongation decreased as the weight percentage of CNC increased. However, the elastic modulus (stiffness) was greatly increased by 34.54% (2594.85 to 3495.19 MPa) in the composite with 1 wt% CNC (Figure 6B). For the YZO–CNC composite, the elastic modulus increased, and the elastic region changed to a straight line instead of a curved line (Figure 6C). In the case of the ZO–CNC composite, the strength decreased linearly, but the slope of the graph did not change linearly according to the weight percentage (Figure 6D).

As described in the above results and shown by the stress–strain curves, linear stiffness and brittleness characteristics were successfully introduced into the composites by the addition of CNC, which is not possible in neat polymers. Consequently, as the plastic region disappeared, only the elastic-region characteristics remained, thus imparting stiffness and toughness characteristics resembling those of the human bones. In particular, the 5:1-wt% ratio of HAP to CNC showed optimal properties for application as a bone model for cutting because the stiffness and toughness of the composite can be changed without reducing the tensile and compressive strengths. In human bones, the tensile strength changes according to both the direction and angle. For example, as the direction changes from longitudinal to transverse, the strength and plastic region gradually decrease because of anisotropy [37]. Moreover, diseased bones tend to become brittle because of the decreased plastic region and exhibit properties such as osteoporosis [38] and osteogenesis imperfecta [39]. Furthermore, knee replacement surgery is performed for osteoarthritis, and the strain energy tends to decrease when such disease occurs [22].

Thus, a harmless composite material that possesses a linear stress–strain curve similar to that of real bone [25] while achieving real femur-bone strength as described above [21] was successfully fabricated and tested herein. Notably, we could also control its strength by adjusting the weight percentage of CNC.

In the case of sample containing only bioceramics, consistent changes in the mechanical property results were observed for each change in the bioceramic weight percentage, but there was no significant difference in the statistical compressive strength and elastic modulus. However, because a consistent change is evident in the case of adding CNC, statistically significant differences are clearly visible in all bioceramic composites through ANOVA statistical analysis.

The measured values of tensile strength, compressive strength, tensile elastic modulus, compressive elastic modulus, toughness, yield strength, and elongation changed with the addition of bioceramics; however, there were no significant differences in the statistical results except for the tensile strength, tensile elastic modulus, and yield strength in the HAP composites, and the tensile strength, compressive elastic modulus, and yield strength in the YZO composites. As shown in Table 3, as the changes in the mechanical properties are considerable in all bioceramic–CNC composite materials, significant differences can be clearly observed in the statistical values.

Among those, and in the elastic modulus results of the tensile tests for all samples, the stiffness increased the most (by 34.54%) in the composites with HAP with added CNC, whereby a statistically significant difference was observed. Furthermore, every sample with CNC demonstrated an increase in the tensile elastic modulus and compressive elastic modulus, and a decrease in toughness (i.e., toughness changed to brittle).

Using a combination of epoxy and CNCs, we aimed to develop a novel artificial bone-like material that would help control various mechanical properties of the artificial bones according to the requirements of bone diseases and bone cutting directions. The existing epoxy-based composite materials exhibit high-strength and non-linear stress–strain characteristics [24]. It is expected that a network-like structure in a CNC-added polymer is capable of matching the mechanical properties of the stiffness and toughness of real bones, which can be adjusted according to the concentration of CNCs in the composite. In other words, a network-like structure can be formed by the like particles around nanocellulose [33]. Since the strength required to achieve cracking can be controlled by adjusting the size of internal particles, this type of epoxy-based composite is expected to have a linear elastic modulus and brittleness similar to that of the real bone [25].

Table 4 summarizes the mechanical properties of the resin-mixed specimens according to the change in the contents of the HAP/YZO/ZO bioceramic filler (in wt%) and nanocellulose (in wt%). The ANOVA results confirmed that all samples fabricated under the same conditions exhibited significant mechanical property changes according to the change in the composition of the bioceramic filler and addition of nanocellulose.

Because ZO has a different crystal phase from that of HAP/YZO, when ZO with a monoclinic phase is mixed with the resin, even a small amount interacts strongly with the matrix, and the mechanical properties do not change according to its weight percentage; therefore, the *p*-value does not show a significant difference [40].

In particular, when nanocellulose is added to HAP, its mechanical properties (i.e., strength, modulus, and toughness) become similar to those of real bone, and there is a significant difference in the *p*-value. In addition, the shape of the stress–strain curve, which indicates a significant change in the mechanical properties, is in good agreement with the change in the linear stiffness. In ANOVA statistical analysis, it has a greater effect on wt% strength and tensile elastic modulus because epoxy demonstrates a small change in compressive strength but a large change in tensile strength. In composite materials, due to the effect of reinforcement, the change in tensile strength is larger than the change in compressive strength. In addition, the changes in the mechanical properties of bioceramic, owing to the wt% of nanocellulose, shows a statistically significant difference, because when CNC is coated with epoxy, the stiffness of the fiber itself is strengthened and the network joint stiffness is also enhanced [33]. Moreover, the fibers widen and the porosity is filled by the fiber network. Therefore, the network density of the composite and stiffness of the epoxy increase. These statistical analysis results confirm that the characteristics of tension show a significant difference.

In summary, we successfully fabricated a femur-bone model that can be used as an educational tool. We estimate that the material cost of a 450 g epoxy sample is approximately 30 USD when using 5 wt% HAP and 1 wt% CNC. Therefore, in addition to reproducing the physico-mechanical properties of the human bone, our formulation is also cost-efficient.

Therefore, it is evident that CNC-added specimens exhibit linear stiffness and brittleness similar to the human bones, as well as morphology characteristics associated with cutting and compression fracture. For example, the size and quantity of the chipped-out pieces are larger than those obtained with pure-polymer–based formulations. This phenomenon is caused owing to the following. When the specimen is cured through the addition of CNC to change the non-linear stiffness and ductility, which are the disadvantages of pure-polymers-based materials, it is crushed while forming a nano-network around the local CNC. In the absence of CNC, networks are formed more densely and are combined to form a network of all specimens such that a plastic region can exist. This is consistent with the tendency of the material to become brittle when solid nanofillers are added to epoxy [34,35]. As the content of nanofillers increases, which cause an aggregation of those nanofiller, stress concentration occurs in the aggregated parts [41].

On rechecking our results, we can confirm that the strength of the synthesized material matches that of real human bone [21]. The results also confirm that the linearity of the elastic region, which is a mechanical characteristic of bone, is maintained [25]. In addition, the results confirm that the change in the particle size after cutting with a saw was changed to mechanical properties similar to bone due to changes in stiffness and toughness with nano network [33] in the SEM result.

Therefore, by adjusting the amount of added CNC, a material with various bone-like mechanical properties, which do not present a safety hazard, can be produced. It is expected that various matrix formation and mechanical properties, which can be applied to various fields, can be obtained through research on adding nanocellulose fibers to more diverse bioceramics.

## 4. Conclusions

In this study, we proposed a bioceramics–epoxy-based composite material (HAP, YZO, and ZO) with cellulose nanocrystal exhibiting linear stiffness and brittleness similar to that of the human bones.

A composite material of the HAP bioceramic and nanocellulose, when synthesized at 5 wt% and 1 wt%, (tensile strength: 32.13 MPa, compressive strength: 125.79 MPa, elastic modulus: 3495.19 MPa.), which are numerically similar to the mechanical properties of real bone. In addition, the linearity of the elastic region, which is the most important property of bone, was maintained in the composite material, due to changes in the stiffness and toughness of the nano network, according to the SEM results. In the subsequent study, we will focus on the network-like nanostructure change in the composite materials through surface treatment of bioceramics with CNC, with an aim to increase the similarity of our material with the real bone.

In the future, this study is expected to serve as a cornerstone for creating an environment where more orthopedic surgeons can practice in a safe and beneficial educational environment, thus facilitating the fabrication of various epoxy-based artificial bones with various mechanical properties.

## Figures and Tables

**Table 1:** Composite materials weight ratio.

Composite Materials	Resin	Hardener	Bioceramic	CNC
Bioceramic 1 wt%	74.25	24.75	1	-
Bioceramic 2 wt%	73.5	24.5	2	-
Bioceramic 3 wt%	72.75	24.25	3	-
Bioceramic 4 wt%	72	24	4	-
Bioceramic 5 wt%	71.25	23.75	5	-
Bioceramic with CNC 1 wt%	70.5	23.5	5	1
Bioceramic with CNC 2 wt%	69	23	5	3
Bioceramic with CNC 3 wt%	67.5	22.5	5	5

**Table 2:** Results of the compression test according to the type of bioceramic–epoxy composite material.

Powder	Weight Percent	Compressive Strength [MPa]	Compressive Elastic Modulus [MPa]
Epoxy	-	120.41 ± 19.25	1536.76 ± 91.43
HAP	1%	125.02 ± 21.57	1396.91 ± 42.92
	2%	109.39 ± 27.85	1583.09 ± 34.96
	3%	132.63 ± 12.46	1599.72 ± 150.37
	4%	133.56 ± 12.96	1556.37 ± 128.70
	5%	139.23 ± 15.61	1473.01 ± 195.34
YZO	1%	131.39 ± 17.39	1402.60 ± 61.59
	2%	130.04 ± 28.64	1406.72 ± 71.12
	3%	132.10 ± 18.46	1450.46 ± 44.84
	4%	137.43 ± 22.69	1613.84 ± 79.85
	5%	112.15 ± 18.46	1285.45 ± 103.97
ZO	1%	75.149 ± 28	1466.73 ± 65.58
	2%	106.43 ± 35.66	1479.09 ± 68.44
	3%	114.33 ± 46.12	1493.41 ± 58.03
	4%	131.50 ± 28.23	1524.79 ± 37.90
	5%	117.83 ± 27.82	1529.30 ± 37.19

**Table 3:** Result of the tensile test by type of bioceramic–epoxy composite material.

Powder	Weight Percent	Tensile Strength [MPa]	Tensile Elastic Modulus [MPa]	Toughness [kJ/m^3^]	Yield Strength [MPa]	Elongation [%]
Epoxy	-	29.63 ± 1.66	1931.98 ± 118.63	962.65 ± 289.36	25.66 ± 0.96	4.30 ± 1.35
HAP	1%	35.10 ± 2.70	2266.89 ± 162.73	953.48 ± 70.29	29.78 ± 1.88	3.62 ± 0.38
	2%	33.75 ± 1.78	2229.62 ± 94.68	759.83 ± 368.83	29.47 ± 1.63	3.09 ± 1.18
	3%	37.22 ± 1.86	2388.49 ± 77.33	833.69 ± 133.41	32.41 ± 1.90	3.06 ± 0.32
	4%	39.39 ± 1.31	2617.47 ± 57.80	849.02 ± 154.76	34.16 ± 1.66	2.97 ± 0.37
	5%	37.29 ± 1.33	2597.85 ± 187.93	689.28 ± 155.16	32.28 ± 1.18	2.62 ± 0.45
YZO	1%	36.06 ± 1.05	2337.50 ± 37.13	876.80 ± 223.92	30.77 ± 0.77	3.35 ± 0.84
	2%	35.56 ± 1.85	2263.95 ± 137.20	911.64 ± 111.33	30.21 ± 0.92	3.42 ± 0.21
	3%	37.00 ± 1.91	2389.97 ± 68.35	839.06 ± 215.80	31.60 ± 1.67	3.08 ± 0.56
	4%	34.84 ± 1.31	2302.19 ± 67.55	735.30 ± 113.88	29.27 ± 0.87	2.95 ± 0.33
	5%	38.24 ± 1.91	2372.80 ± 130.17	740.02 ± 135.55	31.55 ± 1.36	2.83 ± 0.30
ZO	1%	40.20 ± 1.39	2480.57 ± 133.21	823.57 ± 273.50	36.88 ± 1.27	2.89 ± 0.71
	2%	38.46 ± 2.31	2464.00 ± 147.75	783.97 ± 293.82	34.62 ± 2.33	2.84 ± 0.75
	3%	39.67 ± 1.85	2366.60 ± 161.64	961.80 ± 109.43	34.67 ± 1.76	3.30 ± 0.29
	4%	40.22 ± 1.20	2504.22 ± 92.83	932.90 ± 114.47	34.56 ± 1.70	3.19 ± 0.31
	5%	38.83 ± 1.16	2559.81 ± 123.21	868.60 ± 153.28	33.66 ± 0.63	3.05 ± 0.50

**Table 4:** ANOVA statistical analysis results (*p* values) of the correlation between changes in mechanical properties as a function of composition of bioceramic–epoxy composites.

Group	df	TS	CS	TM	CM	T	YS	E
HAP wt%	4	0.00	0.18	0.00	0.09	0.34	0.00	0.20
YZO wt%	4	0.04	0.55	0.26	0.00	0.40	0.02	0.35
ZO wt%	4	0.34	0.14	0.27	0.31	0.63	0.06	0.64
HAP with CNC	3	0.00	0.00	0.00	0.00	0.00	-	0.00
YZO with CNC	3	0.00	0.00	0.00	0.01	0.00	-	0.00
ZO with CNC	3	0.00	0.00	0.11	0.00	0.00	-	0.00

Note: Significant difference at *p* ≤ 0.05. Tensile strength, TS; Compressive strength, CS; Tensile modulus, TM; Compressive modulus, CM; Toughness, T; Yield strength, YS; Elongation, E.

## Data Availability

The data presented in this study are available in insert article.
